# Add Germany to the List—Adventive Population of *Trissolcus japonicus* (Ashmead) (Hymenoptera: Scelionidae) Emerges in Germany

**DOI:** 10.3390/insects12050414

**Published:** 2021-05-04

**Authors:** Christine Dieckhoff, Sophie Wenz, Maura Renninger, Anne Reißig, Helmut Rauleder, Claus P. W. Zebitz, Jana Reetz, Olaf Zimmermann

**Affiliations:** 1 Center for Agricultural Technology Augustenberg (LTZ), Neßlerstr. 25, 76227 Karlsruhe, Germany; helmut.rauleder@ltz.bwl.de (H.R.); jana.reetz@ltz.bwl.de (J.R.); olaf.zimmermann@ltz.bwl.de (O.Z.); 2 Institute of Phytomedicine, University of Hohenheim, Otto-Sander-Str. 5, 70593 Stuttgart, Germany; wenz-s@web.de (S.W.); claus.zebitz@uni-hohenheim.de (C.P.W.Z.); 3 Stuttgart State Museum of Natural History, Rosenstein 1, 70191 Stuttgart, Germany; maura.renninger@smns-bw.de; 4 Arbeitsgemeinschaft bäuerliche Landwirtschaft (AbL, Working Group for Peasant Agriculture) Regional Association Saxony/Thuringia, Zur Burgmühle 1, 99869 Nessetal OT Haina, Germany; reissig@agrarnuetzlinge.de

**Keywords:** *Trissolcus*, egg parasitoid, *Halyomorpha halys*, BMSB, invasive species, biological control, horticulture

## Abstract

**Simple Summary:**

The brown marmorated stink bug, *Halyomorpha halys*, is an invasive pest species of global economic importance. It has a very broad host range and causes severe damages in agricultural, horticultural, and fruit crops. Control measures, including available chemical and mechanical options, have often proved insufficient. Surveys of natural enemies in the newly invaded areas have also shown that natural biological control is generally too low to control *H. halys* populations in the long run. In its native Asian range, egg parasitoids in the genus *Trissolcus* play an important role in controlling *H. halys*. Since the mid-2010s, adventive populations of *Trissolcus japonicus*, a dominant egg parasitoid of *H. halys* in Asia with a narrow host range, have been reported from several countries with prior establishment of *H. halys*. Here, we report the first discovery of *T. japonicus* in Germany. This finding corroborates a northbound expansion of the range of *T. japonicus* in Europe, along with *H. halys*.

**Abstract:**

The brown marmorated stink bug, *Halyomorpha halys*, is a polyphagous pest species of worldwide economic importance. Since the mid-1990s, it has invaded and become established in various countries outside its native Asian range. In the newly invaded areas, biological control by native natural enemies has been shown to be insufficient in the long-term control of this severe pest. Adventive populations of *Trissolcus japonicus*, an important biological control agent of *H. halys* in Asia, have been reported from North America and some European countries since the mid-2010s. This egg parasitoid species seems to follow in the wake of the establishment of *H. halys* populations outside their native Asian range. Here, we report the first discovery of an adventive population of *T. japonicus* in Germany. In 2020, adult *T. japonicus* were recovered from parasitized *H. halys* egg masses (naturally laid and sentinel egg masses) and collected in ruderal areas using an insect suction sampler. The arrival of *T. japonicus* in Germany, unintentional through pathways yet unknown, corroborates a northbound expansion of its range within Europe. Further field surveys will show the extent of its dispersal and establishment capacities within this new distribution area.

## 1. Introduction

The brown marmorated stink bug, *Halyomorpha halys* (Stål) (Hemiptera: Pentatomidae), is a severe pest insect of global economic importance. Originally from East Asia, it became established in North America in the mid-1990s [1,2] and several European countries in the mid-2000s [3,4,5]. Recently, it became established in Russia, Abkhazia, and Georgia (2016) [6], as well as South America (2017) [7]. In Australia and New Zealand, this insect has been intercepted on several occasions, but no established populations have been detected so far [8,9]. In Germany, *H. halys* was first recorded near Lake Constance in October 2010 as well as in Bremerhaven during import inspections whereupon the individuals were instantly eradicated [10]. In subsequent years, *H. halys* populations spread along the Upper Rhine Valley and were obviously transported into urban areas over long distances [11]. The distribution of *H. halys* in Germany has been monitored since 2018 as part of the federally funded monitoring project ProgRAMM [12] and the collected data are also part of a modeling approach for climate-sensitive invasive species [13].

*Halyomorpha halys* is a highly polyphagous pest with over a hundred known host plant associations and can cause severe economic losses in various important fruit, horticultural, and agricultural crops in the newly invaded countries [14,15]. Chemical control strategies have proven to be insufficient in controlling this pest and cannot be considered a sustainable solution in the long term anyway [2]. Physical control measures, e.g., exclusion nets or attract-and-kill strategies, are being tested and hold great promise in minimizing the damage caused by this pest [16,17,18].

Biological control is a central component in the long-term suppression of pest species. In the invaded areas, natural enemies have been shown to be ineffective in controlling *H. halys* populations as parasitism and predation rates are typically under 10% [19,20,21,22]. While several egg parasitoid species have been reported to readily attack *H. halys* egg masses, they either cannot successfully develop in this new host or exhibit highly variable parasitism levels [22,23,24,25]. In its native range, *H. halys* populations are controlled by several predatory and parasitoid species of which egg parasitoids in the genus *Trissolcus* play an important role [26]. *Trissolcus japonicus* (Ashmead), also referred to as the “samurai wasp”, is an oligophagous egg parasitoid of the brown marmorated stink bug [27,28,29]. In its native Asian range, it is the dominant biological control agent of *H. halys* with recorded egg parasitism rates of up to 80–90% [30,31]. Based on this significant impact on *H. halys* populations in its region of origin, *T. japonicus* has been considered as a candidate for classical biological control programs against *H. halys* both in the United States and Europe [19,32]. Recognizing the severe economic impact *H. halys* would cause on its primary industries, New Zealand’s EPA approved the release of *T. japonicus* subject to strict conditions prior to the establishment of *H. halys* populations [33]. So far, *H. halys* has been intercepted numerous times at the border but is not yet known to be established in New Zealand. In Italy, where *H. halys* already caused severe economic losses in fruit crops over the past years, mass production and release of *T. japonicus* were approved by the Italian government in 2020 [34].

Regardless of these efforts, adventive populations of *T. japonicus* have been reported from several countries since the mid-2010s. In 2014, live *T. japonicus* were recovered from sentinel egg masses placed in a wooded habitat as part of a survey of native egg parasitoids in Maryland, USA. Established populations of *T. japonicus* have since been confirmed in 13 U.S. states, Canada, Italy, and Switzerland [35,36,37,38,39,40]. While it is unknown how these adventive parasitoid populations arrived in their new territories, it is likely to assume that the pathways of entry were similar to those of their host, *H. halys*. In Italy, a second adventive *Trissolcus* population, *T. mitsukurii* (Ashmead), was also found parasitizing naturally laid *H. halys* egg masses [37].

Here, we report the first discovery of an adventive population of *Trissolcus japonicus* in Germany.

## 2. Materials and Methods

Between April and October 2020, a field survey regarding the native natural egg parasitoid guild of *H. halys* was conducted in the central Upper Rhine Valley and northern Wuerttemberg, Germany, as part of routine research activities at the LTZ Augustenberg, Karlsruhe, Germany. Survey methods included (a) collection of naturally laid egg masses in natural habitats as well as horticultural, agricultural, and fruit crops; (b) suction sampling of various habitats near known established *H. halys* populations; and (c) placement of sentinel egg masses in the vicinity of a fruit orchard where *H. halys* was known to be present.

### 2.1. Field Surveys

Known host plants of *H. halys*, including both wild hosts and cultivated species, were surveyed for naturally laid egg masses, e.g., *Catalpa* sp., *Paulownia tomentosa* (Thunb.) Steud., *Juglans* sp., *Phaseolus* sp., *Prunus* spp., *Helianthus annuus* L., *Nicotiana tabacum* L., *Glycine max* (L.) Merr., *Cucumis sativus* L. Egg masses collected in the field were then reared individually in 40 mL glass vials (80 mm × 30 mm) under controlled conditions in a climate chamber at 24 ± 2 °C and 16:8 L:D to assess parasitism and emergence rates. Parasitism rates were calculated per egg mass. The number of parasitized eggs was divided by the total number of eggs per egg mass. Here, all parasitized eggs were included in the calculation, irrespective of whether live parasitoids emerged or not. Emergence rates were also calculated per egg mass. The number of successfully emerged parasitoids (always one adult parasitoid per parasitized egg) was divided by the total number of parasitized eggs. A drop of honey was added to each vial for the parasitoids’ sustenance. All emerged parasitoids were counted and sexed, and parasitoids emerged from an egg mass were transferred into 95% EtOH for later morphological and molecular identification.

Parasitoids found guarding *H. halys* egg masses in the field were also collected and stored in 95% EtOH for later identification.

### 2.2. Suction Sampling

From August to September 2020, preferred habitats of *H. halys* were sampled at three locations in Heidelberg (Germany) using the ‘Vortis’ insect suction sampler (Burkard Manufacturing Co. Ltd.). This period is considered as the 2nd oviposition period of *H. halys* in Central Europe [41]. Sampling locations in Heidelberg were in a mixed vegetable and fruit growing area. Locations 1 and 3 were within a bean field (*Phaseolus* sp.) and separated by about 190 m. Location 2 was in a diverse ruderal area between cultivation fields with a distance of about 840 m to Location 1 and 890 m to Location 3. Suction sampling time was set to 1 min. Afterward, each sample was transferred to a zip lock bag and immediately put into a freezer at −20 °C. In the laboratory, the material was transferred to a petri dish (20 cm diameter) and separated by parasitoid superfamilies under a stereomicroscope. Specimens belonging to the superfamily Platygastroidea, particularly specimens with the habitus of Scelionidae, were stored in 1.5 mL vials with 70% EtOH for further analyses.

### 2.3. Sentinel Egg Mass Survey

From mid-August to mid-September 2020, *H. halys* egg masses collected from a laboratory colony were placed as sentinels at six locations on the LTZ experimental fruit farm in Karlsruhe, Germany. The *H. halys* colony was established from field-collected individuals and reared in a greenhouse compartment in net cages (30 cm × 30 cm × 30 cm) stocked with green beans and sunflower seeds at 25.8 ± 4.8 °C and 51 ± 13.5% R.H. at the LTZ in Karlsruhe. Regular card stock, paper tissues, and potted bean plants were placed in the cages as oviposition materials. Fresh (<24 h old) *H. halys* egg masses were collected from the rearing cages and cold stored in a standard refrigerator at 8 °C for at least ten days prior to being placed in the field as sentinels, following the method outlined by Wong et al. [42]. A total of 21 cold-stored *H. halys* egg masses and an additional five fresh egg masses were placed as sentinels in the field. Sentinel egg masses were placed on wild host species in the marginal strips of the LTZ fruit farm, left there for three to four days, and then reared under controlled conditions (as described above) in the laboratory until parasitoid emergence.

All parasitoids of the genus *Trissolcus* were identified morphologically using the keys by Talamas et al. and Tortorici et al. [43,44]. In addition, a subsample of emerged individuals underwent molecular analyses. Voucher specimens are currently deposited in the diagnostic reference collection at the LTZ and additional specimens will also be deposited at the Stuttgart State Museum of Natural History and the State Museum of Natural History in Karlsruhe, Germany.

### 2.4. Molecular Analysis

A molecular analysis was performed to confirm the morphological identification of selected individuals. Two to three legs were removed from every individual in order to extract DNA. For this purpose, the QIAamp DNA Mini Kit (QIAGEN GmbH) was used following the manufacturer’s instructions. The region of the second internal transcribed spacer (ITS2) gene was amplified using the primer pair ITS2-A (5′-TGT GAA CTG CAG GAC ACA TGA-3′) and ITS2-B (5′-GGT AAT CTC ACC TGA ACT GAG GTC-3′) [45]. ITS2 is a widely used and well-established marker in molecular systematics. As ITS sequences are length-variable regions, a threshold of at least 98.5% was used for alignment of the obtained sequences with a known reference sequence of *T. japonicus.* This approach ensured the proper assessment of character homology between the sequences and consequently the correct molecular identification of specimens, in addition to the parallel provision of a morphological identification.

A 25 µL reaction volume was used to perform the polymerase chain reaction (PCR) according to the HotStarTaq Master Mix Kit (QIAGEN GmbH): 6.5 µL sterile water, 12.5 µL Taq DNA polymerase, 2 µL forward primer, 2 µL reverse primer, and 2 µL isolated DNA. The following cycler program was used: In advance, the reaction mixture was heated to 95 °C for 15 min to denature the DNA. This was followed by 38 cycles of 94 °C for 30 s (denaturation), 50 °C for 40 s (annealing), and 72 °C for 60 s (elongation). Finally, the reaction mixture was kept at 70 °C for 10 min to completely elongate the DNA. The PCR products were checked by gel electrophoresis. For Sanger sequencing [46], the PCR products were cleaned with the aid of the Wizard PCR Clean-Up System (Promega GmbH) following the manufacturer’s instructions. A total of 20 µL reaction batch was prepared according to the BigDye Terminator v.1.1 Cycle Sequencing Kit (Thermo Fisher Scientific Inc.). Two sequencing reactions (each with forward and reverse primers) were set up for each sample. The following cycler program was used: first 96 °C for 1 min, then 25 cycles of 96 °C for 10 s, 50 °C for 5 s, and 60 °C for 120 s. The cleaning system DyeEx 2.0 Spin Kit (QIAGEN GmbH) was used to remove the excess fluorescence-labeled dideoxynucleotides (ddNTPs). For capillary electrophoresis in the sequencing machine, 10 to 15 µL HiDi Formamide (Thermo Fisher Scientific Inc.) was added to 5 to 7 µL of the purified sequencing reaction product. The sequence matrix was analyzed using the Sequencing Analysis Software (Thermo Fisher Scientific Inc. Waltham, MA, USA). Further edits were made with the DNASTAR Lasergene Software (DNASTAR Inc. Madison, WI, USA). In a final step, all sequences were checked for their similarity with a reference sequence from *T. japonicus* reference material provided by Dr. Tim Haye (CABI) and the available references in the GenBank (NCBI) using the Basic Local Alignment Tool (BLAST).

## 3. Results

### 3.1. Field Surveys

In 2020, a total of 145 *H. halys* egg masses were collected on various wild and cultivated host plants in the field, and 90 out of the 145 egg masses (62%) were not parasitized. Five of those egg masses did show signs of predation caused by a chewing predator, with predation rates averaging 41 ± 19% (±S.E.) and ranging from 3.7 to 100% of the eggs being predated in an egg mass. Twenty-five egg masses did not produce any live nymphs as nymphal development was arrested for unknown reasons at some point during the developmental phase. In the remaining ones, seemingly unparasitized egg masses, eggs hatched into nymphs with hatch rates ranging from 4 to 100% and an average of 70 ± 5% (±S.E.). Parasitoids were found on or near eight of those egg masses that did not show signs of parasitism when reared in the laboratory. Individuals found guarding an egg mass and successfully recovered in the field were identified as *Trissolcus semistriatus* Nees (four females) and *T. basalis* (Wollaston) (three females). On two egg masses, four and two individuals, respectively, were recovered simultaneously.

Out of the 145 egg masses collected in the field, 55 (38%) were parasitized with parasitism rates ranging from 11 to 100% (average of 92 ± 3%) and emergence rates ranging from 0 to 100% (average 61 ± 5%). A total of 10 egg masses, with parasitism rates of 11% (one egg mass), 25% (one egg mass), and 100% (eight egg masses), were parasitized but did not produce live individuals. One of those egg masses was guarded by two parasitoids which unfortunately escaped in the field. Five indigenous egg parasitoid species were reared from *H. halys* egg masses: *Anastatus bifasciatus* Geoffrey (six egg masses total), *Telenomus* sp. (two egg masses total), *Trissolcus basalis* (eight egg masses total), *T. cultratus* Mayr (two egg masses total), and *T. semistriatus* (one egg mass total). In addition, the exotic egg parasitoid species *T. japonicus* was recovered from a total of 34 egg masses. From nine egg masses, two different egg parasitoid species were recovered from the same egg mass in the following combinations: *A. bifasciatus*–*T. japonicus* (with a ratio of 3.1:1), *Telenomus* sp.–*T. semistriatus* (1.25:1), *Telenomus* sp.–*T. japonicus* (1:26), *T. basalis*–*T. japonicus* (1:16.4). Parasitism rates by *T. japonicus* alone, i.e., an egg mass that produced no parasitoid species other than *T. japonicus*, averaged 100%, and emergence rates were on average 80.9 ± 5.1%. Thirty-one out of the thirty-four naturally laid egg masses parasitized by *T. japonicus* were recovered from a single location with a single cultivated host (*Phaseolus* sp.) on three separate dates (Table 1). At the recovery site, large numbers of adult *H. halys* and *Nezara viridula* L. were aggregated to feed and oviposit on the bean plants. This *Phaseolus* sp. plot was surrounded by a variety of cultivated crops (e.g., tomato, cabbage, lettuce) and wild host plants. 

In addition to the recoveries of *T. japonicus* from *H. halys* egg masses, this species was also reared from naturally laid *N. viridula* egg masses. At site # 2 (Heidelberg area), a total of 27 *N. viridula* egg masses were collected, 7 of which were also parasitized by *T. japonicus* in conjunction with a second species (*T. basalis* (five egg masses) or *T. semistriatus* (two egg masses)). Parasitism rates of these egg masses were all 100% but emergence rates averaged only 63.10 ± 5.9%, for both parasitoid species combined. At site # 1 (LTZ), 17 naturally laid *N. viridula* egg masses were collected with 1 egg mass, recovered from *Helianthus annus*, being parasitized by *T. japonicus*. Here, the total parasitism rate was again 100% with a total of 65.71% parasitoids emerging from the egg mass. In each of these cases, the egg mass was also parasitized by a second, indigenous species—*T. semistriatus* (two egg masses) and *T. basalis* (six egg masses). The indigenous egg parasitoid species was the dominant species emerging from the egg mass in all of these cases. On average, *T. basalis* and *T. semistriatus* emerged from 33.7 ± 8.6% (range 14–62%) and 44.7 ± 19.5% (25–64%) of the parasitized eggs, respectively, compared to 10.9 ± 4.2% (1–24.3%) and 17.2 ± 1.4% (16–19%) in the case of *T. japonicus*.

### 3.2. Suction Sampling

A total of 52 suction samples were taken at three locations in Heidelberg, Germany. Overall, this resulted in a total of 923 individuals in the order Hymenoptera of which 211 individuals belonged to the superfamily Platygastroidea. Ultimately, 11 individuals were identified as *T. japonicus*, 30 individuals were identified as *T. basalis*, and seven individuals were identified as *T. semistriatus* (Table 2). In addition, the genus *Telenomus* was also detected.

### 3.3. Sentinel Egg Mass Survey

A total of 26 sentinel egg masses were placed on wild host plants at the LTZ in Karlsruhe, Germany. Seven of those sentinels were lost in the field, primarily due to predation. Upon retrieval of the egg masses, eggs had clearly been eaten by a predator with chewing mouthparts (as evidenced by the ragged edges of the remaining egg shells; in one case, the card stock was also chewed on, indicating predation by a small rodent), or the card stock was empty, suggesting that the egg mass was either entirely consumed or had fallen off. None of the fresh egg masses were parasitized and of the remaining 15, 2 egg masses were parasitized and produced live parasitoids (Table 1). From one egg mass (28 eggs total), placed on *Cornus* spp. on 1 September 2020, 22 female and one male *T. japonicus* emerged while five eggs did not produce live offspring. In a second egg mass (28 eggs total), placed on a wild shrubby host plant on 11 September 2020, 82% of the eggs (23/28 eggs) were parasitized from which 18 female and one male *T. japonicus* emerged and four parasitized eggs did not produce live offspring. This egg mass was also guarded by one female *A. bifasciatus*, but no live offspring of this species emerged from the egg mass.

### 3.4. Morphological and Molecular Identification

**Figure 1:**
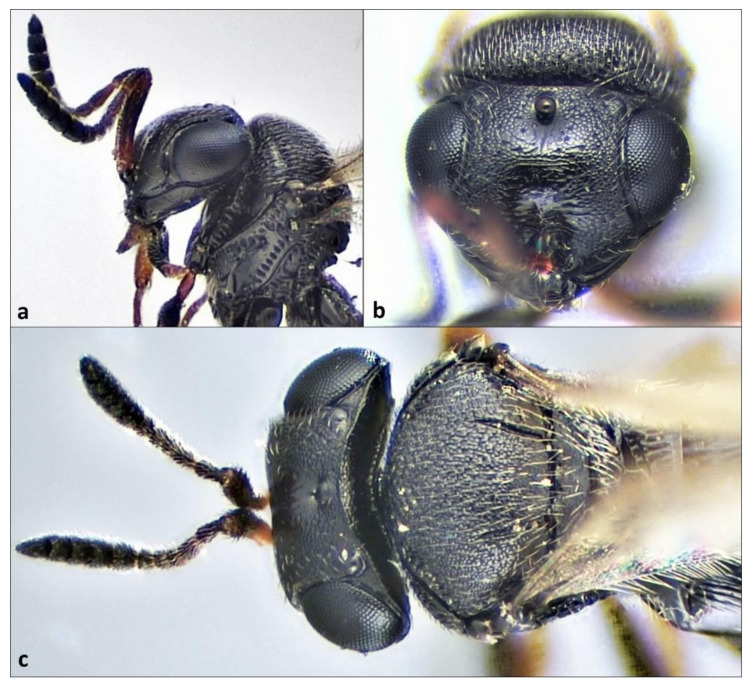
Morphological characteristics of *Trissolcus japonicus* female: (**a**) head, mesosoma, lateral view; (**b**) head, frontal view; (**c**) head, mesosoma, dorsal view.

The specimens of *T. japonicus* recovered from *H. halys* egg masses in this field survey are fully in line with the species concept presented by Talamas et al. [43]. The following morphological characteristics were present in the examined specimens, as described in Talamas’ key to *Trissolcus* of the Palearctic region [43]: inner margin of the orbital furrow smooth and orbital furrow expanded at its intersection with the malar sulcus, vertex between lateral ocelli with hyperoccipital carina uniform and robust, clypeus with four setae, laterotergite 1 without setae, frons above antennal scrobe with transverse rugae, and mesoscutum with notauli present (Figure 1a–c).

The molecular analyses confirmed the morphological identifications. DNA was successfully extracted from eight of the total 11 individuals collected with the suction sampler in Heidelberg. All individuals were at least 98.5% identical to a reference sequence provided by Talamas et al. [47]. These sequences have been submitted to NCBI (Table 3).

## 4. Discussion

Adventive populations of *Trissolcus japonicus* were reported from North America and in Europe in the late 2010s. Subsequent monitoring efforts have been documenting the spread of these populations in those areas [35,38,39,48,49]. What is common to them all is that *T. japonicus* seems to follow in the wake of the establishment of *H. halys* populations outside their native Asian range. Moreover, *T. japonicus* has spread to 13 states across the United States alone since its first discovery in 2014, demonstrating impressive dispersal capacities on the part of the parasitoid [34]. In Europe, considering the discoveries of *T. japonicus* first in Switzerland followed by Northern Italy and now Germany, the dispersal range of this species appears to be expanding north following prior establishment of *H. halys*. At this point in time, it is premature to conclude that the discovery of *T. japonicus* in Germany constitutes an established population. It is, however, highly likely that adult *T. japonicus* were able to survive this past winter and that future additional sampling at the 2020 discovery sites and additional ones will confirm this species’ establishment in Germany.

Ongoing research in North America and Europe has been looking into the fundamental and realized host range of *T. japonicus*. While various laboratory no-choice and choice tests concluded that the host range of *T. japonicus* does include some species within the family Pentatomidae, these tests also confirmed the pronounced preference for *H. halys* egg masses [29,50]. A recent field study conducted in North America confirmed the narrow host range of *T. japonicus* under natural conditions and reported significantly higher levels of parasitism of *H. halys* egg masses compared to those of three native stink bug species, further indicating that *T. japonicus* shows a strong preference for *H. halys* [51]. Studies investigating fundamental and realized host ranges of candidate biocontrol agents typically focus on reproductive effects such as parasitism and emergence rates on target and non-target host species. Hepler et al. [52] applied molecular forensics to assess the impact of mortality caused by non-reproductive effects, e.g., host feeding or aborted parasitoid development. They showed that the combined impact of reproductive and non-reproductive effects of a parasitoid on non-target species may be considerable depending on the host species and the evolutionary history of parasitoid and host species. Taking both effects into account, they found a higher than expected impact of *T. japonicus* on *Podisus maculiventris* in field-exposed sentinel egg masses. It is yet unclear what consequences such parasitoid–host interactions may have at the population level of both the parasitoid and potential non-target host species. Field surveys of the realized host range of *T. japonicus* in Europe have also since been initiated in Italy and Switzerland. These surveys will provide further insights in the years to come. By incorporating molecular forensics into current risk assessment methodology, it remains to be seen which, if any, non-target host species face population-level impacts caused by the presence of *T. japonicus* in Germany.

Haye et al. [29] reported the acceptance of *N. viridula* egg masses by *T. japonicus* in the laboratory under no-choice conditions, but those parasitized eggs failed to produce live offspring. In this study, low-level parasitism of naturally laid *Nezara viridula* egg masses by *T. japonicus* was observed in the field. Each egg mass was also parasitized by another, native *Trissolcus* species at a higher rate compared to parasitism by *T. japonicus*. It is conceivable that successful parasitism of *N. viridula* eggs by *T. japonicus* became possible in eggs previously parasitized by the native parasitoid species. A similar observation has been made for *T. cultratus*, which was able to successfully attack *H. halys* eggs under certain conditions when the egg had already been parasitized by *T. japonicus* [53]. Another explanation for the observed parasitism of *N. viridula* eggs by *T. japonicus* is that *T. japonicus* may have parasitized unviable eggs. The occurrence of unviable eggs in a stink bug egg mass is not uncommon (CD, pers. observation) and the lack of immunological defenses in those eggs may have facilitated parasitism by *T. japonicus*. Future surveys are needed to determine the extent of parasitism of *N. viridula* egg masses by *T. japonicus*. *Nezara viridula* is also an invasive pest species causing severe economic damages in horticultural crops, e.g., cucumber, tomato, and eggplant, in Germany. As is the case with *H. halys*, chemical control of *N. viridula* is insufficient and alternative plant protection strategies are currently under investigation to control this pest species.

So far, biological control by native egg parasitoid species has been shown to be insufficient in long-term control of *H. halys* populations in the invaded areas [20,21,54]. As previous laboratory and field studies have shown, many indigenous parasitoid species are unable to successfully develop in the exotic host’s eggs [22,24,54]. Here, in the case of indigenous female parasitoids found guarding an *H. halys* egg mass but the egg mass not showing signs of parasitism or not producing live adults when reared in the laboratory, unsuccessful parasitism may have caused the abortion of nymphal development. As eggs that did not hatch into nymphs were not dissected in the laboratory, the individual eggs’ fates can only be assumed. While unsuccessful parasitism by native egg parasitoid species still constitutes a form of biological control of *H. halys* in the broadest sense, such parasitism events of essentially unsuitable hosts present what has been termed an “evolutionary trap” [24]. Such evolutionary traps may have a negative effect on native stink bug parasitoid communities caused by parasitism of the unsuitable *H. halys* egg masses [24,52,53]. Immunological defenses of the developing stink bug embryos prevent successful parasitism by native egg parasitoid species. However, parasitism by native species was shown to be successful when eggs were killed by freezing, thus apparently disabling the embryos’ defenses [23,54]. An exception is *A. bifasciatus*, a polyphagous egg parasitoid that also parasitizes lepidopteran eggs, among others, and was shown to successfully parasitize live *H. halys* eggs in a consistent manner in Switzerland and Italy [55]. Stahl et al. [56] assessed the potential of *A. bifasciatus* as a candidate for an augmentative biological control program and concluded that the observed parasitism rates of *H. halys* egg masses in the field were not high enough for the long-term suppression of *H. halys* populations. The establishments of adventive populations of *T. japonicus* in the areas previously invaded by *H. halys* are an important contribution towards the development of integrated management strategies against this economically important pest.

For this study, the ‘Vortis’ suction sampler has proven to be a useful collection method to quantitively sample parasitoid Hymenoptera. Individuals were fully intact after sampling, which was essential for morphological analyses, and the sampler may also be used for live sampling and subsequent laboratory tests with egg masses. Comparative studies showed that very small arthropods can be caught by suction sampling [57,58], which is crucial considering the effort of finding a single species about 2 mm in size. This selective sampling method does not collect larger specimens such as Lepidoptera, bees, and locusts. This minimizes the negative sampling impact on insect diversity, and subsequent microscopic identification of the samples is more efficient due to reduced amounts of by-catch.

## 5. Conclusions

Here, we report the first discovery of an adventive population of *Trissolcus japonicus* in Germany. Naturally collected and sentinel egg masses of *H. halys* showed high levels of parasitism in the new area, thus confirming the strong preference of the oligophagous egg parasitoid *T. japonicus* for its co-evolved host *H. halys* and reiterating its narrow host range. This finding of an adventive population of *T. japonicus* in Germany also corroborates a northbound expansion of its range within Europe, along with *H. halys*. Further field surveys will show the extent of its dispersal and establishment capacities within this new distribution area. Its arrival in Germany, albeit unintentional through pathways yet unknown, constitutes another valuable opportunity to document the realized host range of this promising candidate biological control agent under field conditions.

## Figures and Tables

**Table 1:** Information on *Halyomorpha halys* egg masses (EM) parasitized by *Trissolcus japonicus* in 2020.

Locality	Type EM	Latitude Longitude	Total No. EM	No. EM Parasitized by *T. japonicus*	Parasitism Rate (%, Mean ± SE) [Range]	Emergence Rate (%, Mean ± SE) [Range]	Sex Ratio (% Female, Mean ± SE )
#1—LTZ	naturally laid	49.002972 8.493707	11	1	100	3.5	100
#1—LTZ	sentinel	49.002972 8.493707	26	2	91.1 ± 8.9 [82.1–100]	82.4 ± 0.2 [82.6–82.1]	95.2 ± 0.5
#2—Heidelberg area	naturally laid	49.439123 8.670197	45	31	100 ± 0	80.8 ± 4.5 [18.5–100]	68.1 ± 4.6
#3—Stuttgart area	naturally laid	-^1^	2	1	100	96.4	96.3

^1^ Not included on the grounds of data protection.

**Table 2:** Number of detected individuals of *Trissolcus japonicus*, *T. basalis*, and *T. semistriatus* collected by suction sampling separated by collection date, location, and habitat.

						Number of	
Collection Date	Location	Latitude	Longitude	Habitat	*T. japonicus*	*T. basalis*	*T. semistriatus*
18.08	1	49.439123	8.670197	*Phaseolus* sp. ^1^	0	3	1
18.08	2	49.432074	8.674356	Ruderal	1	1	1
20.08	1	49.439123	8.670197	*Phaseolus* sp. ^1^	3	15	1
03.09	1	49.439123	8.670197	*Phaseolus* sp. ^1^	4	8	0
03.09	3	49.438794	8.667604	*Phaseolus* sp. ^1^	3	3	4

^1^ Cultivated.

**Table 3:** Results of using the Basic Local Alignment Tool (BLAST) (NCBI) of eight *Trissolcus japonicus* specimens collected in Heidelberg, Germany.

			NCBI BLAST		
Collection Date	Species	Accession Number	Query Cover (%)	Identity (%)	Reference Accession Number
18.08	*T. japonicus*	MW781819	99.6	98.5	MN613495.1
20.08	*T. japonicus*	MW781820	99.8	99.8	MN613495.1
03.09	*T. japonicus*	MW781821	98.3	98.5	MN613495.1
03.09	*T. japonicus*	MW781822	98.5	99.8	MN613495.1
03.09	*T. japonicus*	MW781823	98.5	99.8	MN613495.1
03.09	*T. japonicus*	MW781824	98.9	99.8	MN613495.1
03.09	*T. japonicus*	MW781825	98.9	99.8	MN613495.1
03.09	*T. japonicus*	MW781826	98.1	99.8	MN613495.1

## Data Availability

The data presented in this study are available on request from the corresponding author.
